# Ultrasound‐guided intercostal block for the management of intercostal neuralgia in pregnant women: Case series and review of the literature

**DOI:** 10.1002/ijgo.70049

**Published:** 2025-03-05

**Authors:** Laura Tascón Padrón, Norah L. A. Emrich, Carolin Schröder, Florian Recker, Brigitte Strizek, Jorge Jiménez Cruz

**Affiliations:** ^1^ Department of Obstetrics and Prenatal Medicine University Hospital Bonn Bonn Germany

**Keywords:** intercostal neuralgia, neuropathic pain in pregnancy, peripheric blocks, real‐time imaging therapy, ultrasound‐guided intercostal block

## Abstract

Intercostal neuralgia (ICN) in pregnancy is a rare condition which, to date, has not been well studied. Due to the lack of evidence, there is not a consensus about effective therapeutic strategies for this condition. The present study investigated the feasibility of ultrasound‐guided intercostal block (UG‐ICB) using ropivacaine for these patients. A total of 17 cases treated at the University Hospital Bonn from May 2017 to September 2024 were reviewed. Patients presenting with severe unilateral chest or flank pain were diagnosed with ICN. UG‐ICB was applied, and all patients reported immediate pain relief, with only two women requiring a second infiltration due to pain recurrence. No serious adverse events were recorded. The application technique can be consulted in the video related to this publication. The present study highlights that UG‐ICB is feasible, safe and appears to be effective for managing ICN during pregnancy. This procedure provides significant pain relief while minimizing risks to the fetus. Reviewing the literature this study group was unable to find qualitative evidence assessing ICN and rib pain in pregnancy, although this condition is thought to be related to pregnancy. Further research is recommended to improve treatment strategies for ICN during pregnancy and bridge the gap between clinical knowledge and evidence surrounding this condition.

**Abbreviations**
CI: confidence interval
ICB: intercostal nerve block
ICN: intercostal neuralgia
MRI: magnetic resonance imaging
NRS: numeric rating scale
NSAIDs: non‐steroidal inflammatory drugs
OR: odds ratio
QUIPS: quality improvement in postoperative pain management project
SD: standard deviation
TENS: transcutaneous electric nerve stimulation
UG‐ICB: ultrasound‐guided intercostal nerve block
URTI: upper respiratory tract infection

## 1 INTRODUCTION

Intercostal neuralgia (ICN) and rib chest pain during pregnancy is a challenging condition that can significantly impact the quality of life of expectant mothers. The prevalence of intercostal neuralgia in the general population is reported to be between 1% and 3%^1^ and it is often related to secondary neurologic nerve affection such as post zoster neuralgia or neuralgia related to malignancies, but also after tissue trauma.^2^, ^3^, ^4^ However, the specific prevalence of intercostal neuralgia in pregnancy is uncertain. Patients report a stabbing, shooting or burning sharp sensation. ICN can be intermittent or constant and typically presents either as a band‐like pain wrapping along the chest and back or in a thoracic dermatomal pattern and limits movement and deep inspiration and can irradiate to other areas like lower back or the flank.^4^, ^5^ Pain may last for a prolonged period and may continue long after the inflicting disease process has subsided.^6^

During pregnancy, intercostal neuralgia may appear as a consequence of mechanical irritation of the intercostal nerves due to the growth of the uterus or increased intra‐abdominal pressure on the diaphragm, being exacerbated by situations like coughing or small local trauma. Additionally, the effects of progesterone and relaxin on the musculoskeletal system increase the mobility of the costovertebral articulations, which can result in atypical pressure on the intercostal nerves.^7^

Due to its low prevalence, the diagnosis of ICN is challenging and primary clinical, based on the characteristic presentation of pain along the intercostal spaces, which may be exacerbated by movement, coughing, or deep breathing. Typically, pain can be triggered by punctual pressure in one isolated intercostal space. A thorough clinical history and physical examination are essential. Imaging studies such as ultrasound or magnetic resonance imaging (MRI) may be utilized if there is a need to rule out other potential causes of pain but can often be inconclusive.^8^, ^9^

Management of intercostal neuralgia in pregnant individuals poses a particularly challenge due to the limitations on the types of interventions based on safety for mother and fetus.

General recommendations for treating this condition include postural and behavioral modifications, local heat application, manual therapy, electrical stimulation and systemic analgesics.^10^, ^11^

Furthermore, intercostal nerve blocks (ICB) have already been validated as a valuable tool in the management of various thoracic and abdominal pain syndromes, including postoperative pain, rib fractures, and chronic neuropathic pain.^12^, ^13^ However, the safety and efficacy of this procedure specifically in pregnant individuals need to be further investigated.

This study aims to assess the safety and feasibility of ICB for managing pregnancy‐associated intercostal neuralgia ICN by evaluating complication rates and patient outcomes. Furthermore, we intend to systematically review and synthesize the current literature on ICN and ICB during pregnancy.

## 2 MATERIALS AND METHODS

Data from medical records from patients being treated with ICB between May 2017 and September 2024 at the University Hospital Bonn, Germany were extracted. This center started offering UG‐ICB for women with intercostal neuralgia in pregnancy from May 2017. Woman presenting with unilateral chest or flank pain were evaluated and diagnosed of ICN if maximal pain can be triggered by applying pressure in one intercostal space. Other conditions related to chest or flank pain such as, for example, pyelonephritis, pre‐eclampsia, cardiac disorder were excluded as necessary. The patients were previously informed about the alternatives to infiltration (physiotherapy, application of heat, oral analgesics such as paracetamol, etc.) and gave their written consent for the performance of ICB with local anesthetics. All patients included gave their informed consent for data collection and analysis and their use for research as is standard in our institution. Approval of the local ethics committee was provided (registration no.: 208/18).

The intercostal nerve block procedure consisted of injection of 5 to 8 mL of a 0.75% ropivacaine solution, into the affected intercostal area under ultrasound guidance.

**FIGURE 1:**
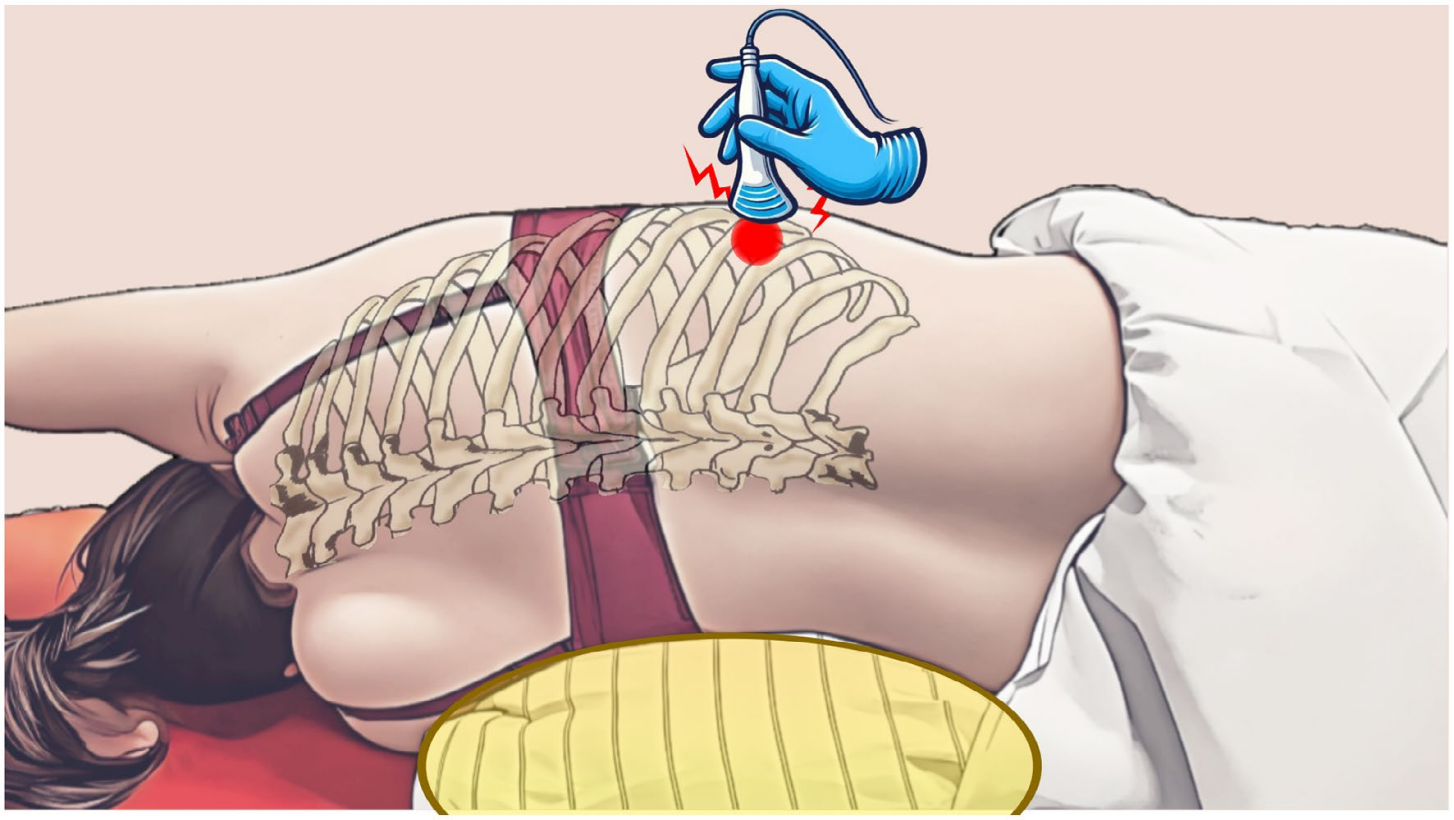
Positioning of the patient to maximize space between the ribs.

Patients were placed in a lateral position, with a pillow under the flank to maximize the intercostal space (Figure 1). After proper positioning, the target intercostal space was identified and marked with a pen. A small skin area was cleaned with antiseptic solution. The ultrasound probe was positioned in alongitudinal orientation, perpendicular to the ribs, along the posterior axillary line. The ribs were visualized as hyperechoic structures appearing as bright,curved lines. The intercostal muscles were identified between the ribs, with the pleura and lung appearing posterior (deeper) to the intercostal space. The intercostal neurovascular bundle is located below the inferior boder of the superior rib forming the intercostal space.

**FIGURE 2:**
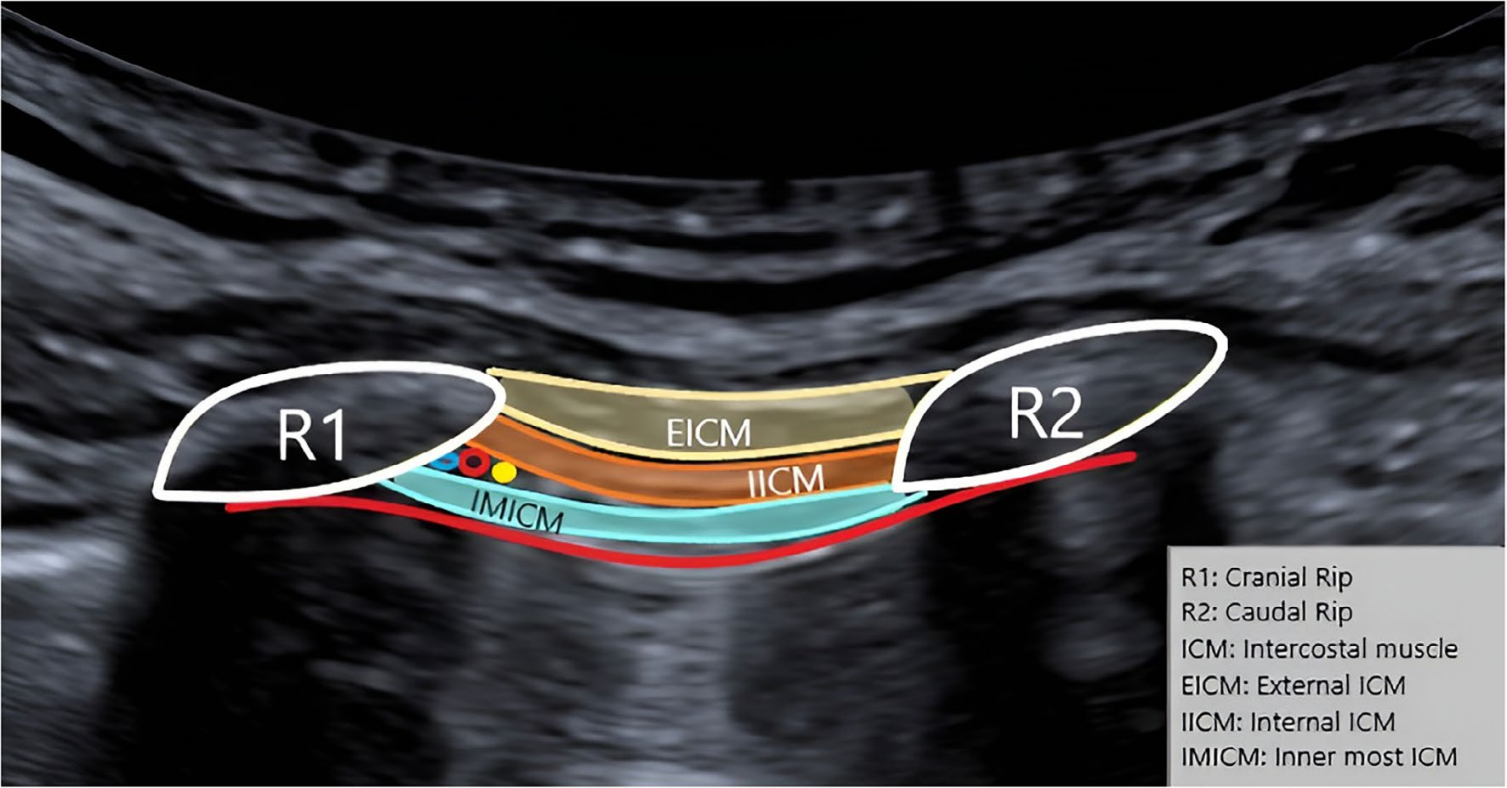
Identification of the different layers of the intercostal space on ultrasound.

**FIGURE 3:**
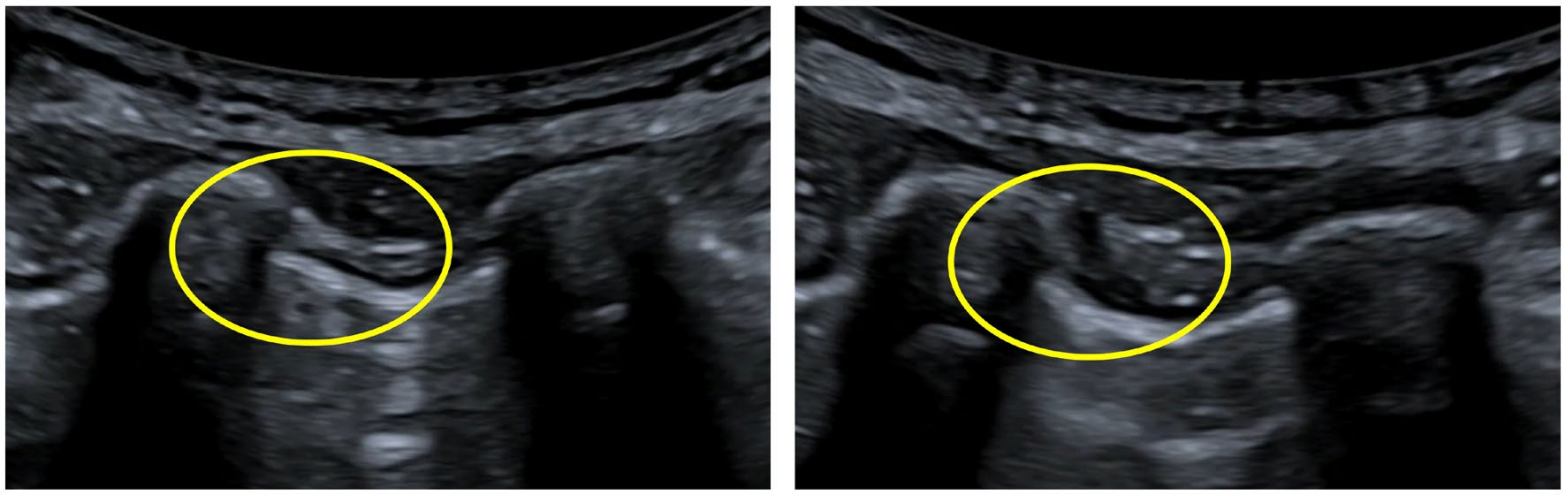
Subcostal space before (left) and after (right) infiltration. Observe deformation of the area.

A 22‐gauge, 50 mm long needle was then inserted in‐plane to the transducer and advanced until the tip was placed just below the inferior border of the rib after passed the fascia of the internal intercostal muscle (see Figure 2). After negative aspiration to confirm that the needle was not in a blood vessel, 6 to 8 mL of a ropivacaine 0.75% solution was injected (see Video S1). Correct application of the infiltration was confirmed by deformation of the subcostal space (Figure 3). The needle was then extracted. Fetal well‐being was checked ultrasonographically after completion of the procedure. Patients were monitored for 30 min after the block has been performed to exclude any complications. Telephonic follow up was performed by all patients 2 days after infiltration. If pain recurrence was reported patients were scheduled for a second infiltration.

## 3 RESULTS

The present study collected data retrospectively from 17 women who presented to the center with severe pain in chest or flank and were diagnosed with ICN and subsequently treated with ICB. The demographic characteristics from each patient are shown in Table 1.

**TABLE 1:** Review of cases of intercostal neuralgia—patient characteristics, week of gestation, time of appearance.

Patient number	Age (years)	Weeks of gestation	Left/right	SCS	Season	Previous URTI	Gravida	Para	Duration of symptoms (days)	Previous therapies	BMI (kg/m^2^)	Need of reinfiltration
1	38	35	L	7	Spring	No	2	2	6	No	31.1	Yes
2	30	26	R	10	Spring	Yes	1	0	7	Corticoids as spray	24.2	No
3	29	30	R	12	Winter	No	1	0	56	Systemic antibiotic^a^	24.2	No
4	32	38	R	10	Summer	No	1	0	1	No	34.4	No
5	31	38	L	11	Autumn	No	3	2	3	Acetaminophen	25.9	No
6	29	38	R	10	Autumn	No	1	1	0	No	24.3	No
7	21	35	R	5	Summer	No	1	0	0	No	34.6	No
8	45	38	L	10	Autumn	No	4	3	0	No	28	No
9	37	36	R	12	Spring	No	4	3	7	No	29.4	No
10	27	33	R	10	Autumn	Yes	1	1	0	No	28.9	No
11	36	29	L	12	Winter	No	4	1	6	No	26.1	No
12	29	19	L	10	Winter	No	1	0	5	Systemic antibiotic^a^	19.9	No
13	31	17	R	9	Spring	No	1	0	2	Systemic antibiotic^a^	26.6	No
14	23	28	L	11	Summer	No	1	0	10	No	22.2	No
15	36	36	R	10	Summer	No	1	0	12	Systemic antibiotic^a^	29.3	No
16	33	34	R	9	Summer	Yes	2	1	7	Acetaminophen Piritramide	31.3	Yes
17	36	24	L	11	Winter	No	4	1	5	Acetaminophen Piritramide	25.2	No

*Note*: BMI, calculated as weight in kilograms divided by the square of height in meters.

Abbreviations: BMI, body mass index; SCS, Subcostal space; URTI, upper respiratory tract infection.

^a^ Systemic antibiotic for suspected pyelonephritis: cephalosporine intravenously or oral.

The mean age of the participants was 31.9 (±5.8) years, while the mean gestational age at the time of ICN was 31.4 (±6.7) weeks. Regarding the body mass index (BMI, calculated as weight in kilograms divided by the square of height in meters), the mean was 27.3 (±4.1) kg/m^2^. In respect to affected area we observed that the right side was more affected (10 patients [58%]) versus seven patients (41.2%) and affection of the lower subcostal space was inferior to rib 10, 11, 12 (13 patients [76.5%]). Seasonal distribution and parity were similar among patients.

Observing the course of the symptoms 12 patients presented in the hospital after having more than 7.5 (±13.0) days of pain. A total of eight patients (47.1%) received no previous therapy before consultation, and five patients (29.4%) were externally treated with systemic antibiotics under clinical suspicion of pyelonephritis.

ICB could be successfully applied in all 17 patients, and all patients reported immediate pain relief after the application of the block. Two patients (11.8%) reported recurrence of pain within 2 days and needed a second infiltration, being pain free after this reintervention.

Regarding side effects, no serious adverse events were registered. Two patients reported metallic flavor suggesting intravasal application, and one patient reported self‐ limiting intensive headache for 5 min after application.

## 4 DISCUSSION

The present study demonstrated that ultrasound guided ICB using ropivacaine in pregnant women is feasible and appears to be safe. Symptom control was achieved in all cases, indicating effectiveness of the intervention. To the best of our knowledge, this is the first study assessing this entity and its therapy in pregnancy.

The evaluation of the demographic factors revealed that the majority of cases affected the subcostal spaces underneath the so called “floating ribs” (spaces below 10th, 11th or 12th ribs), which are more mobile thus they are not connected to the sternum. This augment of mobility makes these subcostal nerves more susceptible to mechanical irritation. The physiological changes related to pregnancy like increase of diaphragmatic pressure, hypermobility of articulation caused by relaxin and the organ displacement due to the growth of the uterus may explain why these areas were more affected.

We aimed to perform a systematic review of the literature assessing ICN and rib pain in pregnancy. Using common MeSH terms like “rib pain” “intercostal neuralgia”, “intercostal block” and “pregnancy”, did not provide more than a couple of results using the usual databases Pubmed, Medline or Web of Science. Furthermore, many of these results include isolated case reports and were published more than twenty years ago.^11^, ^14^ As a contrast performing a similar research using the same terms in a standard web‐based search engine like Google delivers many recommendations from physiotherapists, midwifes and medicine‐specialized web portals. This illustrates a relevant gap between clinical evidence and regular practice.

ICN pain is bothersome and can cause a relevant impairment and loss in the quality of life of pregnant women. Due to its low incidence, there is a lack in consensus of recommendation for the management of ICN during pregnancy. The existent treatment strategies consist of non‐pharmacologic or pharmacologic interventions. Some cases would need hospitalization and use of oral or intravenous analgesics. In non‐pregnant population conventional treatment of ICN includes non‐steroidal inflammatory drugs (NSAIDs), opioids, antidepressants^15^ or anticonvulsants.^16^ Due to the known side effects and fetotoxic effects of these drugs during pregnancy, other drugs such as paracetamol are preferred, which is known to have a lower analgesic effect for neuropathic pain.^17^

Topical lidocaine patches have been identified as a safe option for managing thoracic neuralgia, including intercostal neuralgia, during pregnancy.^7^ These patches can provide palliative relief for the pain associated with intercostal neuralgia without posing significant risks to the mother or the developing fetus, but reduction of the symptoms is achieved later.

Nonpharmacologic interventions such as cooled radiofrequency ablation have been suggested as potential treatment options for intercostal neuralgia in the general population.^18^, ^19^ This procedure has not been validated for pregnant women, so other conservative less invasive strategies such as transcutaneous electric nerve stimulation (TENS) are preferred.^20^

Neurectomy, which involves the surgical removal of the affected intercostal nerve, has been proposed as a treatment option for intercostal neuralgia in non‐pregnant individuals with chronic pain.^4^ However, this intervention is very invasive and should be reserved for individual cases since risks and benefits of such surgical interventions during pregnancy need to be carefully evaluated.

Since intercostal pain is described as neuropathic pain, application of local anesthetics seems to be a logical approach. The application of local anesthetics is well studied in different scenarios in gynecology and obstetrics. This includes the treatment of persistent vulvodynia, transversus abdominis plane blocks following lower transverse laparotomies, pudendal block during labor or port site infiltration before laparoscopy,^21^ and so on. Interestingly the pain relief, particularly in chronic pain conditions, lasts beyond the expected effect attributable to its pharmacokinetic characteristics. This mechanism remains inadequately understood. For example Arner et al.^22^ concluded that local anesthetic conduction blocks can exhibit effectiveness beyond the expected duration, with complete pain relief lasting up to 48 h and further relief persisting for 4–6 days.^23^ Specific properties such as water solubility, strong penetrability, and high protein binding ratio^24^ and the direct application of the affecting point^25^ are characteristics that may contribute to the sustained action of these agents, leading to extended pain relief.

For specially complicated cases with long lasting pain the use epidural catheters have been described.^11^ It is well known that regional anesthesia is a safe option for other conditions during pregnancy. ICB is easy to perform and only few contraindications are described in the literature.^26^

ICB complications such as pneumothorax, intraneural injection or bleeding are rare, and if performed with ultrasound guidance significantly reduced.^26^ The use of ultrasound guidance has improved safety and efficacy of peripheral block procedures by allowing for real‐time visualization of the relevant anatomical structures and precise delivery of the local anesthetic.^27^, ^28^, ^29^

The present study had some limitations. First, the presented cases were retrospectively selected after indication of therapy so no comparison could be performed with patients receiving other treatments. Due to this, conclusions regarding effectiveness of the therapy should be carefully interpreted. Nevertheless, the data presented show that this procedure is feasible and secure for pregnant women. The low number of patients included in this study could also be considered a limitation. As already previously assessed, prevalence of this condition is whether low or underestimated so it is no possible to know the actual prevalence of ICN in pregnancy. This is in line with the lack of existing literature that we confronted when preparing this review.

## 5 CONCLUSION

UG‐ICB is a feasible and secure technique for therapy of ICN during pregnancy. Patients included in the case series reported immediately pain relief with very low rates of recurrence. Using this local approach allows to treat intense neuropathic pain without exposing the fetus to systemic therapies. Further research is needed to understand this condition and to optimize pain management in pregnancy.

## AUTHOR CONTRIBUTIONS

LTP: Conceptualization, data curation performing the investigation, writing original draft and editing. CS: Data acquisition and review of the manuscript. NLAE: Data acquisition and review of the manuscript. FR: Project administration and supervision, writing review and editing. BS: Project administration and supervision, writing review and editing. JJC: Data validation, conceptualization, formal analysis, development of the methodology, writing, reviewing and editing the manuscript. We confirm that the manuscript has been read and approved by all named authors and that there are no other persons who satisfied the criteria for authorship but are not listed. We agree to be personally accountable for our contributions to this literature, for both their accuracy and integrity. Questions regarding accuracy and integrity have been investigated, resolved and the resolution documented in the literature. The authors read and approved the final manuscript.

## CONFLICT OF INTEREST STATEMENT

The authors report no conflict of interests.

## Supporting information


**Video S1.** Explained demostration of UG‐ICB.

## Data Availability

The data that support the findings of this study are available on request from the corresponding author.
